# Supplementary material for: Efficient Activation of Peroxymonosulfate by Biochar-Loaded Zero-Valent Copper for Enrofloxacin Degradation: Singlet Oxygen-Dominated Oxidation Process
Source: Nanomaterials (Basel). 2022 Aug 18;12(16):2842. doi: 10.3390/nano12162842 (PMC9415348; doi:10.3390/nano12162842)
Supplement: Supplementary file 1 [file nanomaterials-12-02842-s001.zip › nanomaterials-1842432-supplementary.pdf]

Supplementary Materials

Efficient Activation of Peroxymonosulfate by Biochar-Loaded Zero-Valent Copper for Enrofloxacin Degradation: Singlet Oxygen-Dominated Oxidation Process

Jiang Zhao [1,†], Tianyin Chen [1,†], Cheng Hou [1], Baorong Huang [1], Jiawen Du [1], Nengqian Liu [1], Xuefei Zhou [1,2,*] and Yalei Zhang [1,2,*]

1 State Key Laboratory of Pollution Control and Resources Reuse, College of Environmental Science and Engineering, Tongji University, Shanghai 200092, China
2 Shanghai Institute of Pollution Control and Ecological Security, Shanghai 200092, China
* Correspondence: zhouxuefei@tongji.edu.cn (X.Z.); zhangyalei@tongji.edu.cn (Y.Z.); Tel./Fax:+86-21-65985811 (Y.Z.)
† These authors contributed equally to this work.

Table S1. Details of the eluents and detection wavelengths of HPLC.

Compound	Eluents	Wavelengths (nm)
Enrofloxacin	acetonitrile: (0.35% phosphoric acid + triethylamine, pH = 3.0) = 17: 83	277

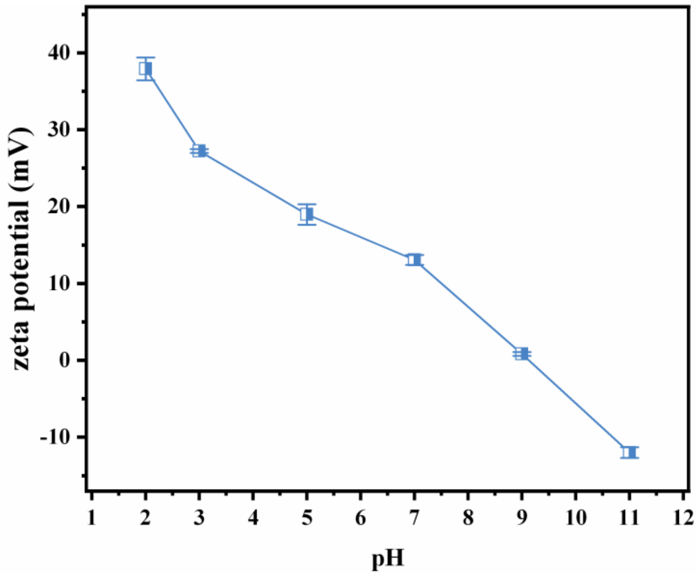

Figure S1. The zeta potential of CuC in different pH.

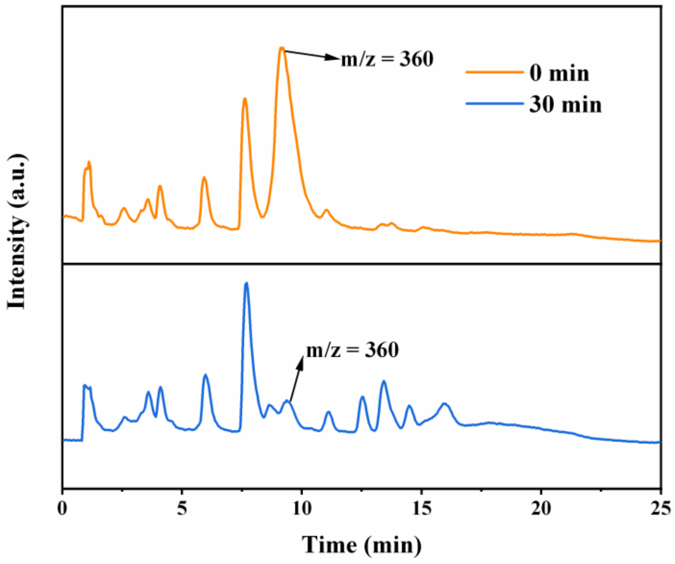

Figure S2. LC-MS chromatogram of 0 min and 30 min.

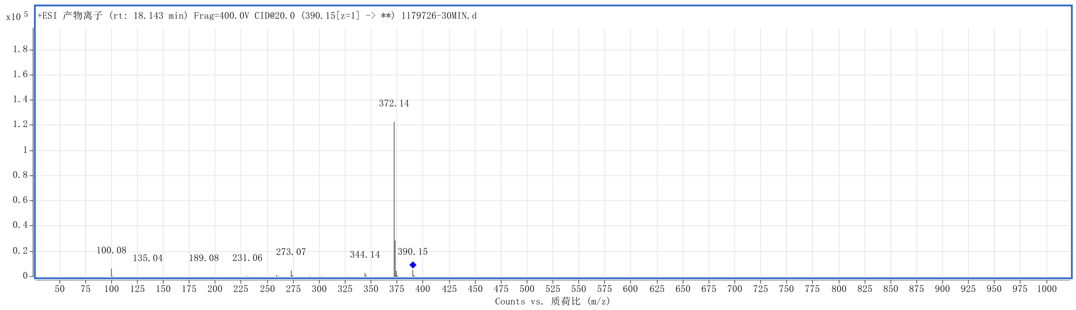

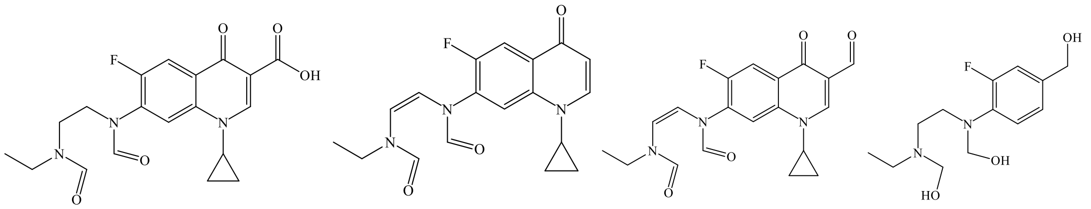

BP1 m/z = 390 **m/z = 344** **m/z = 372** **m/z = 273**

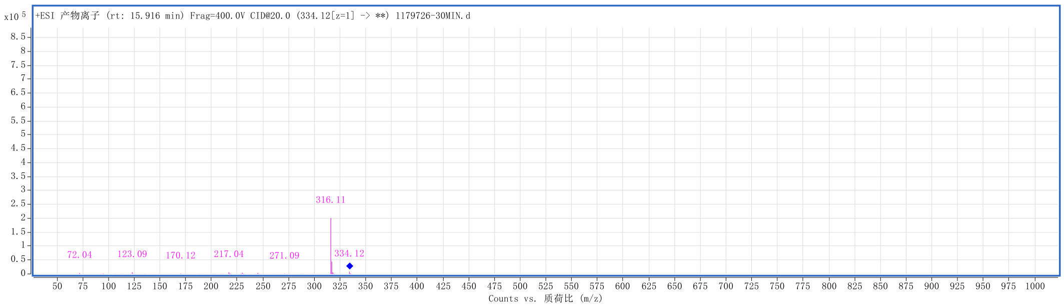

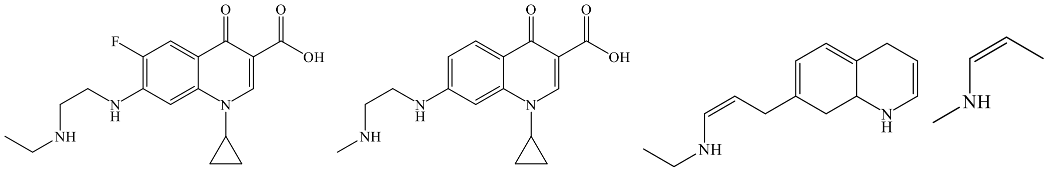

BP2 m/z = 334 **m/z = 316** **m/z = 217** **m/z = 72**

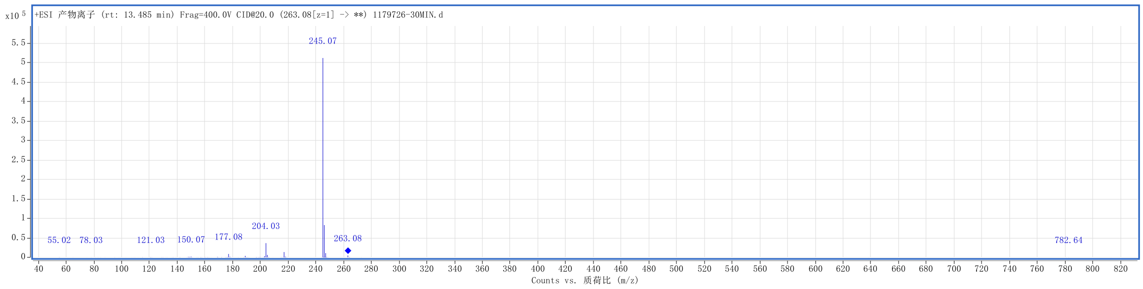

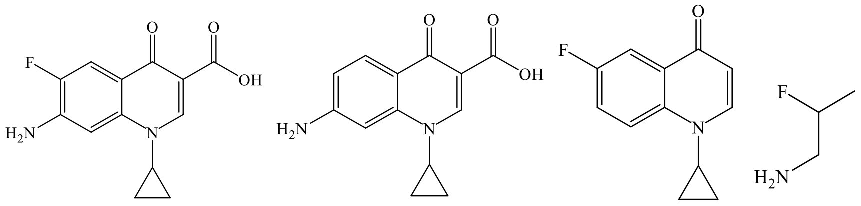

BP3 m/z = 262 **m/z = 245** **m/z = 204** **m/z = 78**

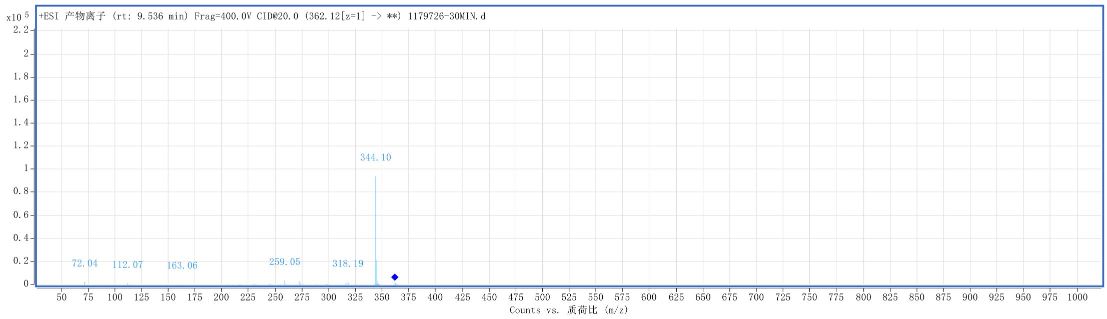

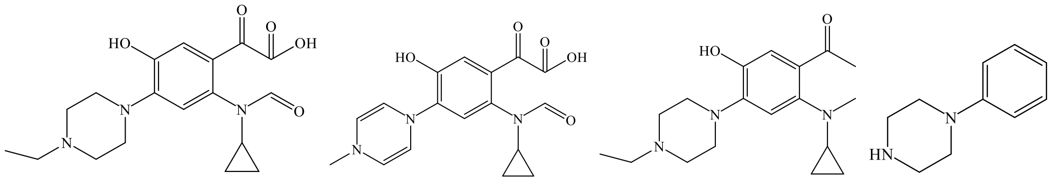

BP4 m/z = 362 **m/z = 344** **m/z = 318** **m/z = 163**

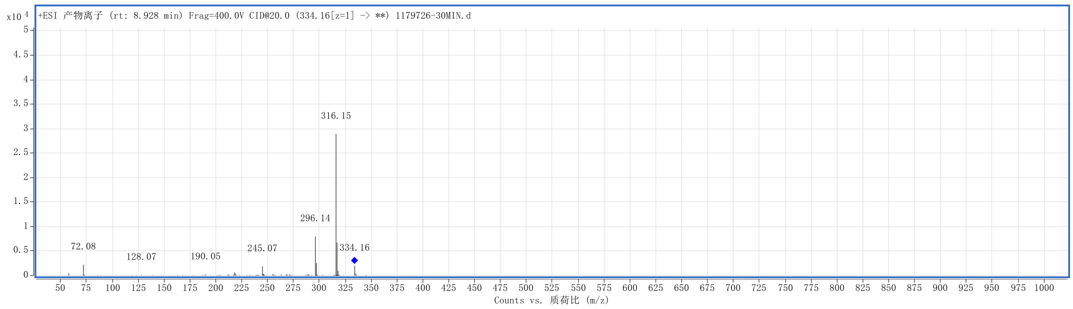

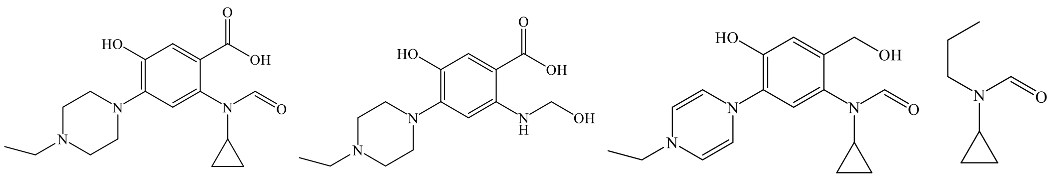

BP5 m/z = 334 **m/z =296** **m/z = 316** **m/z = 128**

Figure S3. Secondary MS spectra of the ENR and the possible identified intermediates.
